# Hypersensitivity to man-made electromagnetic fields (EHS) correlates with immune responsivity to oxidative stress: a case report

**DOI:** 10.1080/19420889.2024.2384874

**Published:** 2024-08-04

**Authors:** Thawatchai Thoradit, Marthe Chabi, Blanche Aguida, Soria Baouz, Verene Stierle, Marootpong Pooam, Stephane Tousaints, Casimir D. Akpovi, Margaret Ahmad

**Affiliations:** a UMR8256, CNRS, IBPS, Sorbonne Université, Paris, France; b Department of Biology, Faculty of Science, Naresuan University, Phitsanulok, Thailand; c Cabinet Medicale, France; d Non-Communicable Diseases and Cancer Research Unit (UR-MNTC), University of Abomey-Calavi, Cotonou, Benin; e Department of Biology, Xavier University, Cincinnati, OH, USA

**Keywords:** Antioxidant therapy, EHS, electromagnetic field sensitivity, electromagnetic fields, EMF - LF, oxidative stress, reactive oxygen species, wifi

## Abstract

There is increasing evidence that exposure to weak electromagnetic fields (EMFs) generated by modern telecommunications or household appliances has physiological consequences, including reports of electromagnetic field hypersensitivity (EHS) leading to adverse health effects. Although symptoms can be serious, no underlying mechanism for EHS is known and there is no general cure or effective therapy. Here, we present the case study of a self-reported EHS patient whose symptoms include severe headaches, generalized fatigue, cardiac arrhythmia, attention and memory deficit, and generalized systemic pain within minutes of exposure to telecommunications (Wifi, cellular phones), high tension lines and electronic devices. Tests for cerebral, cardiovascular, and other physiological anomalies proved negative, as did serological tests for inflammation, allergies, infections, auto-immune conditions, and hormonal imbalance. However, further investigation revealed deficits in cellular anti-oxidants and increased radical scavenging enzymes, indicative of systemic oxidative stress. Significantly, there was a large increase in circulating antibodies for oxidized Low-Density Lipoprotein (LDLox), byproducts of oxidative stress accumulating in membranes of vascular cells. Because a known primary effect of EMF exposure is to increase the concentration of cellular oxidants, we propose that pathology in this patient may be causally related to a resulting increase in LDLox synthesis. This in turn could trigger an exaggerated auto-immune response consistent with EHS symptoms. This case report thereby provides a testable mechanistic framework for EHS pathology with therapeutic implications for this debilitating and poorly understood condition.

## Introduction

Electromagnetic hypersensitivity (EHS) is a serious, often debilitating condition resulting in a diverse array of adverse health symptoms in individuals exposed to man-made electromagnetic fields (EMFs). Highly disparate symptoms may include sleep disorders, asthenia, headaches, memory loss, difficulties in concentration, dizziness, musculoskeletal pain, acute and chronic inflammation, gastrointestinal disorders, skin conditions and mood disorders. These occur in the presence of EMF emitted by various devices, including mobile phone base stations and handsets, Wi-Fi routers, DECT telephones, household appliances, compact fluorescent and halogen light bulbs, power lines, power transformers, or smart meters [1,2]. These devices moreover induce symptoms at far below current reference exposure levels [3–6]. As a consequence, EHS cases may be forced to discard personal electronic devices and avoid highly exposed areas such as shopping centers, public transportation or even hospitals. Some have resorted to wearing EMF-shielding clothes, and living in isolated areas distant from sources of EMF exposure such as countryside, woods and caves [2]. Available epidemiological data points to increasing numbers of cases of EMF sensitivity, ranging from 1.6% (Finland) to 10.3% (Germany) in European countries, for example [2]. Because EMF devices are virtually ubiquitous in the modern world, EHS syndrome detracts significantly from the quality of life and productivity of these individuals.

Despite the increasing numbers of affected people and the possibility of becoming a significant public health issue, the existence of EHS still remains controversial. There are no clear criteria by which to define it, and it remains a self-diagnosed condition with no standardized organic pathological signs, and extremely variable symptoms and severity. A related problem is that there is as yet no proven pathogenetic mechanism for EMF hypersensitivity, and therefore no consensus regarding diagnostic criteria and treatment options. Indeed, much of the literature is plagued by controversy and contradiction, as well as the absence of standardized EMF exposure and measurement protocols (see e.g. [7]). Accordingly, the World Health Organization has classified EHS as a “disabling condition” consisting purely of “non-specific symptoms that lack apparent toxicological or physiological basis or independent verification” and have “no clear diagnosis criteria” [8]. Unfortunately, this definition denies any causal relationship of EHS symptoms to EMF exposure, which is in contradiction to virtually all of the self-reported patient case reports to the contrary. The continuing controversy and lack of WHO recognition has had the result that EHS is often neglected by the medical community or simply written off as a psychosomatic (imagined, psychotic) disorder unrelated to EMF exposure. This attitude has badly discouraged research into EHS. In fact, up until today no validated therapies are available and there has been a marked lack of progress in understanding either the etiology or underlying mechanisms involved [1,2].

Recently, there has been a breakthrough in our understanding of the fundamental mechanisms by which human cells respond to EMFs. In particular, many labs have now shown that EMFs induce rapid increase in cellular-free radicals and reactive oxygen species (ROS) and thereby enhance cellular oxidative stress. This has been demonstrated in human cell cultures as a response to low-frequency ELF-MF [9–11] as well as to high-frequency RF exposure in the MHz and GHz range [12–14]. These effects moreover occur rapidly (within minutes of exposure), and at low, subthermal signal amplitudes found in the man-made environment which are all well below safety reference-level guidelines (see e.g. [10,11,14]). In sum, human cell exposure to EMF seems to induce a mild increase in intracellular oxidants and ROS (reactive oxygen species). This is thought to result in part from spin chemical (quantum physical) mechanisms which modulate the reaction rates of cellular redox active flavoenzymes such as cryptochromes or mitochondrial enzymes [10,11,15,16]. Thus, exposure of human cells to even extremely weak electromagnetic fields found in the man-made environment can trigger measureable and reproducible fluctuations in intracellular ROS.

The interest from a public health standpoint comes from the many and varying effects of ROS and oxidative stress on cellular function and disease [17]. At high concentrations or under prolonged chronic exposure conditions, increasing the concentration of cellular ROS can cause oxidative damage to cellular lipids, proteins, and DNA with ultimately mutagenic and pathological consequences [1718^18^. Indeed, excessive or chronic increase in cellular ROS results in a condition known as oxidative stress, that over time can promote health problems including inflammation, acute or chronic pain, cardiac and circulatory problems, nausea, difficulties with concentration and memory, and promotion of the onset of aging [17]. It should be emphasized however that potential harmful effects of man-made EMFs to which humans are exposed have been extensively and exhaustively studied and do not induce measurable pathology in the general population at exposure levels considered safe in international guidelines [3,5]. Thus, EMF exposure does not produce deleterious symptoms in the general population, likely due to efficient cellular anti-oxidant and detoxification mechanisms.

However, it has been suggested that persons with enhanced sensitivity to ROS or oxidative stress, perhaps resulting from defective cellular anti-oxidant mechanisms, may be particularly intolerant to even modest EMF exposure levels [11,14]. In support of this idea, past reports have shown a correlation between cellular markers for oxidative stress and cases of self-reported EHS (see e.g. [1] and references therein). As oxidative stress is not among the usual tests performed in patients reporting EHS symptoms, this may be a promising new avenue to find standardized diagnostic criteria and/or underlying causes and treatment options.

Here, we present the case report of a 25-year-old male with acute self-reported symptoms of EHS. Multiple prior testing had revealed no physical abnormalities and he did not respond to any therapeutic intervention. His case baffled medical specialists who were inclined to disavow the truthfulness of his symptoms and attribute them to psychosomatic manifestations. However, testing specifically for markers for oxidative stress showed many anomalies, including significant increases in antibodies against LDLox, which are lipid oxidation products induced by oxidative stress. These results are discussed with respect to possible underlying mechanisms of EHS disease progression and toward developing novel and effective therapeutic interventions.

## Materials and methods

### Patient evaluations, laboratory tests, and blood tests

All patient evaluations and test results reported in this case report were prescribed by the patient’s primary care physician and conducted by appropriately accredited biomedical laboratories and in-hospital medical personnel. The physical symptoms were collected by medical personnel and the primary care physicians, not self-reported by the patient. The data presented in this manuscript cover the complete medical history of the patient over the last 4 years.

The analyses were performed by the following entities:

Electrocardiogram (ECG) analysis, thoracic evaluation, blood calcium-level evaluation, blood enzymatic tests for transaminases aspartate aminotransferase (ASAT) and alanine aminotransferase (ALAT), Gamma-Glutamyl Transferase (Gamma GT), creatine phosphokinase (CPK), Troponin I ultrasensitive, hematology, cryoglobulin analysis, autoimmune antibody analysis, glycemia, circulating thyroid-stimulating hormone (TSH) level, renal sufficiency tests, and allergen sensitivity tests were all performed by Celas Cerballiance Corporation, located at 387 Ave Octave Butin 60,280 Margny Les Compiegne, France.

Vascular pathology by Supra-aortic artery trunk echo color Doppler was performed and evaluated by cardiologists at the Cabinet d’Angéiologie Les Cedres, located at 2 BIS Avenue du Libération 60,200 Compiegne, France. Magnetic resonance imaging (MRI) was performed by the Service d’Imagerie Médicale at the Centre Hospitalier Intercommunal, located in Compiegne, France. The gastric examination was conducted at the Centre Médical de Pathologie, located at 96 bis, rue Saint Joseph 60,200 Compiegne, France. Analyses of blood serum levels of antioxidants including Vitamins A, C, E, co-enzyme Q10, and beta-carotene as well as enzymatic markers of oxidative stress such as superoxide dismutase (SOD) and GSH peroxidase were performed by Biocome Laboratoire, Laboratoire Saint Come, 9 rue Jean Jacques Bernard 60,200 Compiegne, France.

All therapeutic interventions were conducted and evaluated by the respective medical specialists and communicated by the physician in charge.

### Ethics approval

The study authors did not at any time obtain samples from the patient, analyze samples, or prescribe treatment for the patient. All testing and data presented in this study were obtained by licensed medical laboratories (Government of France). The tests and treatments presented in this report were approved and prescribed by the patient’s general practitioner, Dr Stephane Tousaints, M.D. (licensed by the Conseil national de l’Ordre des Médecins, France). Medical records were obtained with patient written approval. The study complies fully with the code of ethics, approved by the Ethics Committee at Sorbonne Universite: https://sante.sorbonne-universite.fr/en/faculty-medicine/regulatory-acts/code-ethics

### LED light illumination treatments

The Red-Light Therapy Photobiomodulation device used for antioxidant treatment (Figure 4) was a 730 nm LED light wrap (https://synlyte.com/product/synlytetm-flw811-neck-custom-led-light-pad/). The device emits light at an intensity of 100 W/m^2^ at the skin surface. It was used according to standardized protocols and has been shown to have both antioxidant and anti-inflammatory properties [19]. The device was obtained from Synlyte SAS (2 Rue du 1^er^ Mai, Palaiseau 91,120 France).

## Results

### Background

The patient is a 25-year-old Caucasian male residing near the city of Compiegne, France, 195 cm, 115 kg, with marked sensitivity to exposure to electromagnetic fields and telecommunications. His symptoms include but are not limited to: sensation of pressure in the skull, sensation of acute and burning cerebral pain following a vascular trajectory; pain in the temporal region and circle of Willis; fatigue; difficulty in concentration; memory loss; lack of focus; reduced motor function; insomnia; nausea; hearing deficit and/or hyperacuity; and problems with vision. In addition, the patient experienced acute thoracic pain, dyspnea, and spasms (although without affecting oxygen saturation values); variations in arterial tension; intestinal transit problems; generalized trembling; spasms/microspasms in internal tissues (not related to muscular activity); and contractile sensations in the teeth. There is further generalized pain throughout the body, heaviness in the limbs, reduced sensitivity/feeling in the face and arms, and generalized retraction in the superficial veins (clearly visible in the hands but also occurring throughout the body).

These exposure symptoms occurred in response to household telecommunications and telecommunications devices in the 2G, 3G, 4G, and 5G bandwidths including Wifi, Bluetooth, and GPS emitters as well as from proximity (less than 50 m) to electrical fields such as high tension power lines. The subject was particularly sensitive to 4G and 5G bandwidths. Many of these EHS symptoms were induced within 20 min of exposure after the subject’s leaving a reduced EMF exposure zone such as his isolated house in the countryside, lacking telecommunications equipment, and electrical appliances. To give a frame of reference for the types of energies involved, emission by a good Wi-Fi signal received at a tablet or cellular device can be as low as −50 to −70 dBm, which translates to about .000001222 Watts/m^2^ received at a two-dimensional planar surface. This contrasts to the European safety standard of 1.6 Watts/Kg total body weight maximum exposure Safety Absorption Rate (SAR) recommended for cell phone devices, which could never be achieved at this intensity [3,6,14]. Details of measurement methods and magnitudes of RF exposure likely to be encountered in daily life are also further outlined in numerous studies [20–24].

Milder symptoms were provoked in the patient by meteorological conditions such as thunderstorms, strong winds and rain. The severity of the symptoms and their persistence after onset varied according to signal proximity and strength. For instance, exposure to his personal portable phone began to induce symptoms in this patient already after 5 min, and became intolerable after 30 min; requiring at least 2 h for recovery. These symptoms became more severe and recovery took longer in cases of multiple exposure to different elicitors, such as occurred during visits to the city, in public transportation, or in hospitals with multiple electrical, scanning and telecommunications equipment.

In addition, the severity of symptoms had progressively increased over time during the last 4 years. For this reason, the patient is now unable to function in a normal workplace environment and resides in an isolated, home with minimal electronic and electrical devices in the countryside, distant from powerlines and radio antennae. It is a debilitating condition which severely affects the quality of life and future professional prospects of the patient as he is unable to function in a regular, EMF exposed work environment without becoming seriously ill. More details on the severity and potential public health consequences of EHS are found in recent reviews (see e.g. [2,7,25]).

## Medical tests

### Tests of organ functioning

Multiple analyses were performed to discover the origin and physiological basis for these symptoms. Since 2020, these tests have included the following: cerebral MRI; Supra-aortic artery trunk echocolordoppler (TSA Echocolordoppler); cerebral angioscan; cardiac echography; EEG (electroencephalogram); Electrocardiogram (ECG), and abdominal echography. All tests were performed by accredited hospitals or Medical Laboratory Test labs and were evaluated by the chief medical specialist in charge (see methods). None of these tests showed significant anomaly.

## Blood analyses and biochemical testing

The patient has further been the subject of rigorous physiological and biochemical testing to detect underlying pathology or infectious disease (see Figure 1a–c for summary of test results 2020–23). A hemogram (HMF) measuring complete blood count (CBC) and erythrocyte sedimentation rate were all within reasonable range of reference values and no significant anomalies were detected (Figure 1a) Similar non-significant results were obtained for hemostasis as determined by levels of prothrombin and other markers for blood ion composition (Figure 1b) Hepatic and renal markers (SGOT, SGPT, Gamma GT alkaline phosphatase, transaminases (ALAT and ASAT), creatine, blood urea levels) proved within reference values with the exception of a moderate increase (25%) in ASAT (Figure 1b). The same was true of blood phosphorus, calcium, plasma globulins, CRP, Creatine Phosphokinase (CPK), Troponin I, Gamma-GT, vitamin B12, vitamin D, folate, ferritin, LDH and thyroid hormones (cortisol, TSH).

**Figure 1c.**
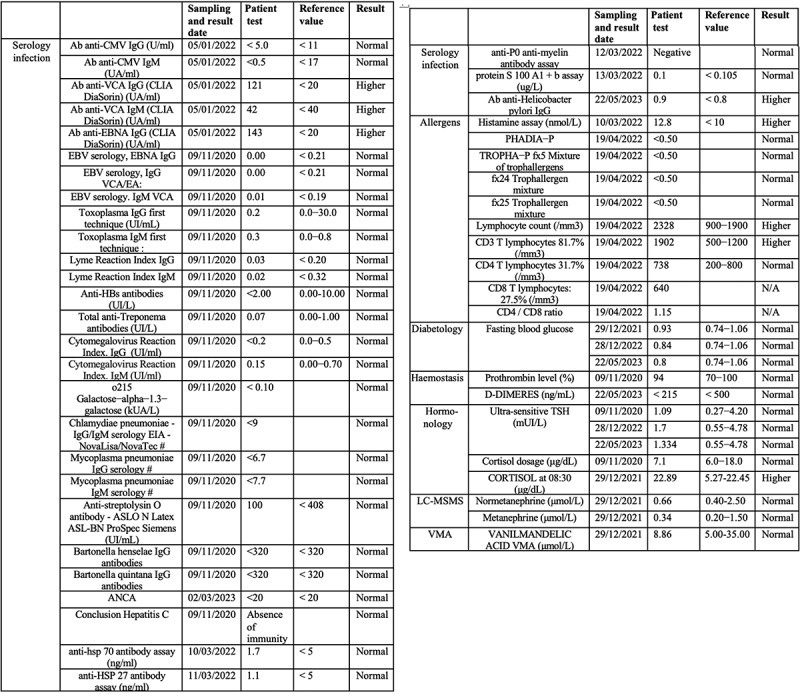
(a–c) Blood test analysis 2020–2023. All analysis was performed by accredited biomedical laboratories and validated by the physician in charge (see methods). Date of test (sampling date), recorded value (test value), values in the normal human range (reference value) and patient outcome (result) are recorded. The result is displayed as normal (reference range), higher (above reference range) and lower (below reference range). a. Hematology analysis. b. Blood biochemistry, serum protein and enzymology analysis. c. Blood tests for antibodies against infectious agents, allergens and allergic reactions, blood glucose and diabetes, hormone tests for thyroid (TSH, cortisol) and kidney function.

Blood glucose levels were within 10% of reference range as was blood insulin, peptide C, cholesterol (total, HDL and LDL) and triglycerides. Catecholamines, metanephrins and Vanilmandelic Acid (VMA) levels were normal (Figure 1c).

**Figure 1a.**
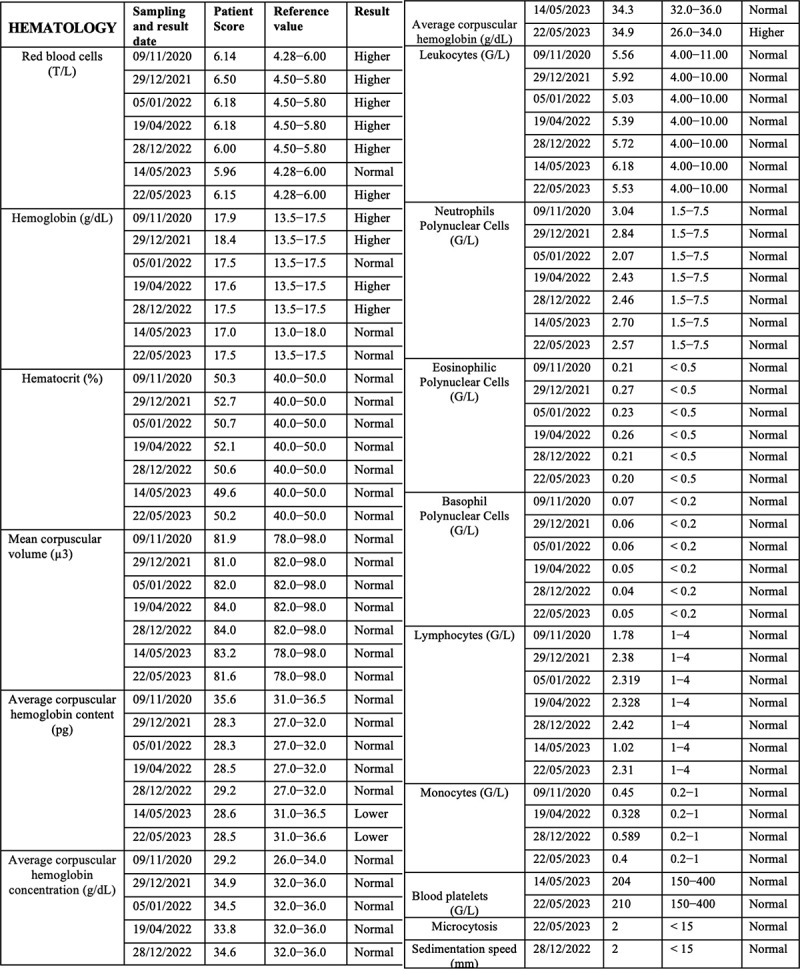

**Figure 1b.**
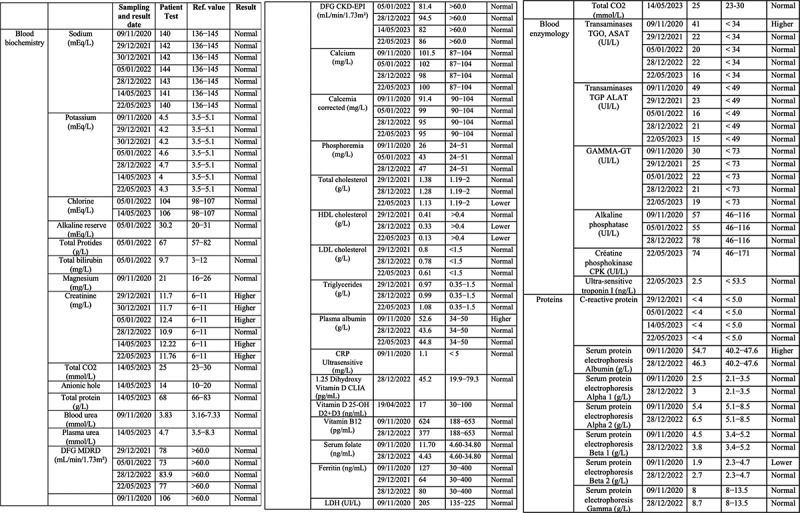

## Allergens, exposure to infection and immunological testing

Serologic tests for prior infectious agents proved positive for Epstein Barr virus, mononucleosis, and mildly positive for helicobacter pylori, but negative for other infectious agents (toxoplasma, Lyme disease, hepatitis B, hepatitis C, HIV virus, syphilis, chlamydia pneumoniae, and cytomegalovirus (CMV)) (Figure 1c). In addition, testing for various immune and allergic conditions was carried out. These included cryoglobulin and Antineutrophil Cytoplasmic Antibodies (ANCA) analysis, Trophatop © allergen analysis, tryptase, Phadiatop © allergen analysis, and lymphocyte analysis (T3/T4/T8 CD3, CD4, and CD8 lymphocytes) (Figure 1c). In sum, although there was evidence of prior infections (Epstein Barr, mononucleosis and helicobacter pylori) and mild increase in T-lymphocyte counts, serological test results were mostly within the reference range and provided no plausible basis for the patient’s pronounced EHS symptoms.

## Oxidative stress: a possible underlying pathology

**Figure 2.**
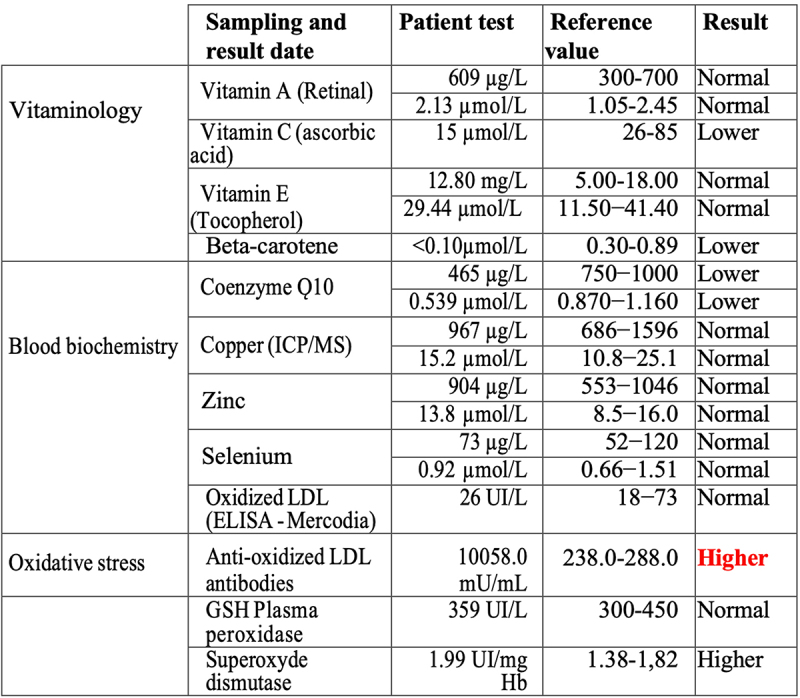
Blood test analysis November 2023 for evidence of oxidative stress. All analysis was performed by accredited biomedical laboratories and validated by the physician in charge (see methods). Type of test (test), patient recorded value (value), values in the normal human range (reference value) and patient outcome (result) are recorded. The result is displayed as normal (reference range), higher (above healthy reference range) and lower (below healthy reference range).

Recent evidence in human cell cultures has shown that a direct effect of exposure to electromagnetic fields is the induction of cellular ROS – which are highly reactive molecules potentially implicated in pathology. Such formation of ROS is triggered both by exposure to telecommunications in the Ghz range [14] and to static or low frequency (10–1000 MHz) ELF-MF magnetic fields [11,14] consistent with this patient’s sensitivity range. We therefore explored the possibility that EHS susceptibility in this patient might be correlated with reduced tolerance for oxidative stress. We obtained measurements of the levels of antioxidants including Vitamins A, C, E, co-enzyme Q10, and beta-carotene as well as of enzymatic markers of oxidative stress such as superoxide dismutase (SOD) and GSH peroxidase. The results from this biochemical analysis of blood samples are summarized in Figure 2.

The patient indeed showed a significant deficit in serum levels of cellular antioxidants Vitamin C, beta-Carotene, and Co-enzyme Q as compared to the norm, together with elevated levels of the ROS scavenging enzyme superoxide dismutase (SOD), indicating the presence of excess ROS. The most striking difference was an approximately 40-fold increase in the concentration of antibodies to oxidized Low-Density Lipoprotein (LDLox) (Figure 2). LDLox is a toxic lipid byproduct of oxidative stress which can contribute to atherosclerosis and inflammation at high concentrations. By contrast, the levels of circulating LDLox measured in the bloodstream were not elevated in this patient (Figure 1b, 2). This suggests that increased levels of LDLox, triggering the formation of anti-LDLox antibody, likely occurred only transiently in the patient, or else are localized in particular organs or cell types.

Both possibilities are consistent with the patient’s EHS symptoms. Even a small and localized increase in LDLox induced by EMF exposure, either in the membranes of the vasculature or in other organs, could provoke a rapid and severe immune reaction, consistent with the rapidity, severity, and nonspecific nature of the symptoms.

## Therapeutic interventions

**Figure 3.**
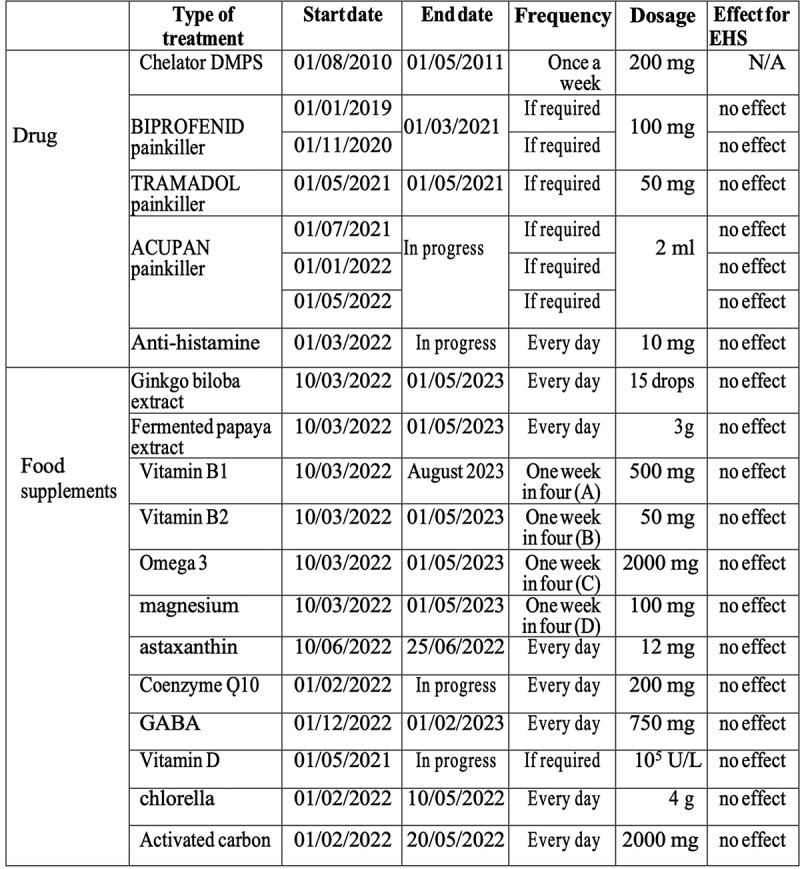
Therapies attempted from 2021 – 2023. Chelation therapy (2010, first entry in table) is included as treatment of heavy metal poisoning of the patient in childhood but not directly relevant to EHS symptoms, as these only became apparent in 2019. The type of treatment, start date, end date, dosage and frequency is presented in the table. Effectiveness was assessed by the patient’s subjective feedback as to improvement of EHS symptoms.

Due to the inconclusive nature of the initial test results (Figure 1a–c), medical interventions in this patient were first limited to mild pain killers, anti-allergenic treatments, and nutritional supplements (summarized in Figure 3). These included prescription of antihistamines and painkillers, as well as fermented papaya (rich in vitamin C) and other supplements, vitamins B1, B2, and co-enzyme Q. None of these interventions provided relief against EHS symptoms. The patient then attempted 1 month of an anti-inflammatory diet eliminating gluten, dairy products, red meat, fried food, refined sugar, lactose or processed meats. The diet consisted mainly of lean meat (chicken, turkey), fresh fruits and vegetables, and whole oatmeal (breakfast). The goal was to eliminate potential inflammatory triggers that could increase internal oxidative stress and therefore sensitivity to EMF. This did not prove effective in preventing EHS symptoms.

**Figure 4.**
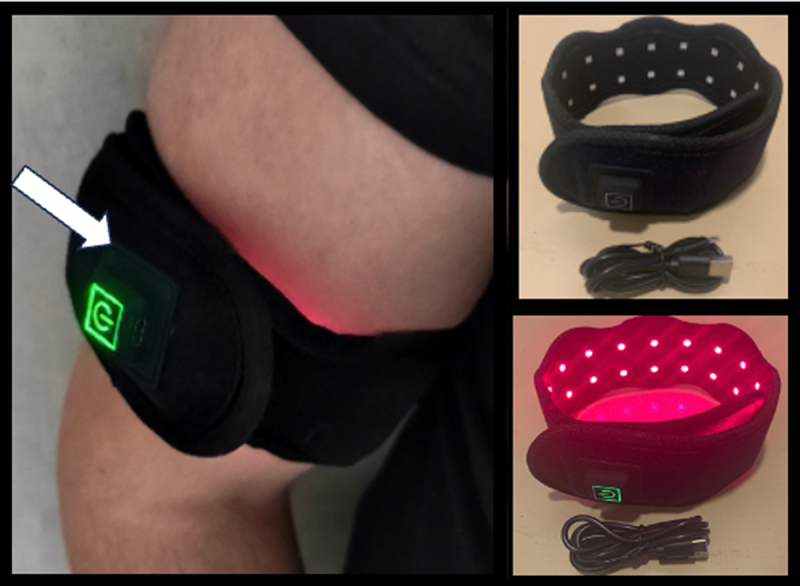
Photobiomodulation Therapy (Nov – December 2023). A LED light wrap emitting infrared light (see methods) was placed around the arm as indicated and illuminated for 5 minutes exposure per day. Light wavelength was 730nm and intensity at the skin surface at 100w/m^2^. The patient reported relief from milder EHS symptoms caused by weather conditions (thunderstorms, strong winds) but not to more severe EHS elicitors (telecommunication, Wifi).

It should be noted in this context that the effectiveness of antioxidant nutritional supplements in general in treating disease related to cellular oxidative stress has not been proven and is currently controversial [26,27]. Therefore, as an alternative to nutritional supplements, the patient attempted Photobiomodulation therapy. This method involves repeated transient exposure to near-infrared (NIR) light from an LED wrap therapy device (Figure 4). Such infrared light-based therapy devices are sold over-the-counter as ‘wellness products’ and have been used for years in the treatment of inflammation, pain relief and to stimulate regeneration/wound healing [28]. Recently, it has been demonstrated that Photobiomodulation therapy works by stimulating an immediate and dramatic downregulation of intracellular ROS in human cells. This occurs through stimulation of anti-oxidant enzymes following an oxidative burst produced by mitochondria in response to stimulation with near-infrared light [19]. Such paradoxical effects of achieving net, long-term decrease in oxidative stress by brief stimulation of transient oxidants is a process known as hormesis, which is a classic feature of cellular redox reactions (reviewed in [17]). Since these anti-oxidant effects can persist for a period of hours or even days [19], we hypothesized that photobiomodulation may prove useful against acute symptoms of EHS.

In a preliminary trial, a small 4 × 7 cm^2^ area of the patient’s skin over the arm was exposed to NIR (near infrared) light using an LED therapy device set at the optimized wavelength of 730 nm (Figure 4). The intensity of 100W/m^2^ at the skin surface was chosen to ensure sufficient penetration to reach underlying tissue and blood vessels (see [19,29]). The small surface area ensured only a mild exposure to the bloodstream, in this way avoiding possible adverse side effects. The patient used the device daily for 5 min over a period of several weeks. He reported no noticeable adverse effects and, to the contrary, experienced protection and relief from milder EHS symptoms resulting from meteorological conditions (thunderstorms and weather anomalies). However, the treatment was not effective for the more severe EHS symptoms, possibly because it was not applied for the correct exposure duration or to the correct anatomical region. Further research taking into account these variables may therefore prove promising.

## Discussion

EHS syndrome has proven exceptionally challenging to characterize due to the wide ranging and nonspecific nature of symptoms, absence of clear diagnostic criteria and biomarkers, and lack of consistency in the reported EMF triggers between different individuals [2]. This has caused skepticism concerning whether the syndrome is causally related to electromagnetic field exposure, and confusion regarding etiology and treatment methods. Nonetheless, certain themes re-occur in the symptoms of self-reported patients, which have included neurasthenia, headache and skin symptoms, thoracic pain, sleep disturbance, fatigue, altered skin capillary flow, altered blood pressure and heart rate, cognitive issues and anxiety. Recurring physiological effects among patients have included inflammation, nitroso-oxidative stress, BBB (blood-brain barrier) disruption/opening and brain neurotransmitter changes. Several of these reactions to electro magnetic fields appear to mimic auto-immune or allergic reactions. Finally, EHS syndrome is also often accompanied by hypersensitivity to other forms of environmental stress, for example multiple chemical sensitivity (MCS) [2,7].

However, the primary mechanisms by which EMF triggers EHS syndrome have been poorly understood and controversial, and there has been no known causal mechanisms. This problem has been compounded by the more general controversy in the field of magnetobiology, regarding the underlying mechanisms by which electromagnetic fields do/do not impact on living systems [15].

Recently, work by our group and others has led to a breakthrough in understanding the primary mechanism by which living cells respond to EMF (weak electromagnetic and sub-thermal radiofrequency fields). These studies have shown that exposure to both low-frequency ELF-MF and telecommunications in the GHz range results in mild cellular oxidative stress, generating a transient increase in cellular oxygen radicals [9,11–14]. A quantum physical (spin chemistry) explanation for these phenomena exists in that even extremely weak magnetic fields can modulate the reaction rates of normal cellular redox reactions. This causes observed increase in the concentration of cellular ROS (reactive oxygen species) in the presence of electromagnetic fields [10,15,16]. Direct proof now exists that transient accumulation of ROS (reactive oxygen species) occurs within minutes of cellular exposure to telecommunications, even at the very low amplitudes of cellular phones and home Wifi [14]. It should be noted that this mechanism also fits very well with past observations of rapid changes in voltage-gated calcium channel (VGCC) activation resulting from RF exposure (see e.g. Pall, 2017), as ROS is a known modulator of intracellular calcium flux [30,31], and is induced in response to many different EMF signals [32,33].

Thanks to these new mechanistic insights, it becomes possible to suggest an underlying mechanism for EHS that is consistent with the present case report findings. We hypothesize that accumulation of intracellular ROS, which in normal individuals is quickly neutralized by cellular anti-oxidant mechanisms, may by contrast do real cellular damage in persons who have reduced anti-oxidant cellular protection mechanisms (see considerations on redox homeostasis and hormesis in [17]). Since EMF exposure specifically triggers ROS formation, such persons could therefore also be at increased risk to suffer EHS symptoms. Consistent with this suggestion, the current case report showed reduced levels of anti-oxidants (e.g. Vitamin C, beta-Carotene, and Co-enzyme Q) in the bloodstream along with elevated levels of superoxide dismutase (a ROS scavenging enzyme), indicative that the person was under oxidative stress consistent with reduced anti-oxidant coping mechanisms. Indeed, the suggestion that EHS symptoms may be linked to increased oxidative stress is not new, and such correlations have been established in multiple studies in the past (e.g. [1]). However, there has been no explanation of how the rapid and often violently debilitating symptoms of EHS could be triggered by the trace accumulation of ROS triggered by EMF, even assuming the person is compromised in anti-oxidant defense mechanisms.

The current case report provides a clue as to the missing link between the dramatic symptoms experienced by EHS patients and a subtle increase in cellular oxidative stress triggered by man-made EMFs. The answer may lie within the immune system, as demonstrated by the increase in this patient’s antibodies to LDL oxidase, a lipid oxidation product formed by reactive oxygen species (ROS) in the cell. Even a minor increase in LDLox, for instance in exposed membranes of the vasculature, could plausibly elicit a severe auto-immune reaction. This would explain the rapid onset and severity of the patient’s EHS symptoms, including to brain, blood vessels and circulation. Furthermore, the progressive nature of his symptoms, which have worsened over time, are consistent with increasing immune response as a result of repeated exposure (‘boosting’) to an EMF elicitor. The fact that standardized tests for autoimmune or allergen pathology of this patient proved negative (CRC, ANCA, etc.) (Figure 1c) is consistent with the transient nature of the EMF elicitor and the rapid elimination of ROS and ROS byproducts subsequent to EMF removal.

The suggestion that EHS may trigger an immune response against cellular by products of oxidative stress is furthermore consistent with past work showing a correlation between EHS and other forms of environmental hypersensitivity. For instance, many patients reporting EHS also have an increased incidence of multiple chemical sensitivity (MCS). Multiple chemical sensitivity causes a violent averse reaction to touching or ingesting trace amounts of common chemicals that are harmless to most people [2]. The symptoms are very similar to those caused by EHS, both debilitating and painful. Like EHS, MCS is correlated with increased intracellular oxidative stress, and could be particularly pronounced in persons with reduced oxidative stress coping mechanisms. We therefore suggest that the trigger for EHS may not necessarily come from excessive electromagnetic field exposure, but instead from other more extreme environmental triggers resulting in production of antibodies to by-products of cellular oxidative stress. Subsequent exposure to electromagnetic fields would then elicit the same symptoms as MCS and by similar mechanisms (i.e. stimulation of ROS and subsequent formation of oxidative stress-related cellular byproducts, in turn provoking an exaggerated immune reaction).

In the present case, the patient did not report MCS. However, he had a family history of heavy metal poisoning which was not caused by excessive environmental exposure, but rather due to genetic susceptibility throughout his close family. The grandmother and her three daughters, which included the mother of the patient, had reduced ability to eliminate heavy metals. This caused significant childhood heavy metal poisoning in all five family members, consistent with reports that heavy metal poisoning often has a genetic component [34]. The heavy metal poisoning was successfully treated and alleviated in all family members subsequent to its diagnosis. Nonetheless, although their blood heavy metal levels returned to normal, these same family members all developed subsequent increased susceptibility to electromagnetic fields (EHS). For example, the present subject of this case report was successfully treated with chelation therapy for heavy metal poisoning at the age of 13, whereas his symptoms of EHS only appeared many years later. Therefore, it is possible that elevated oxidative stress or other pathology induced by this early heavy metal poisoning may have triggered his later sensitivity to EMF exposure.

It is important to emphasize that we do not claim here to prove this mechanism, nor even to provide proof that EHS is indeed caused by exposure to electromagnetic fields. Generalized conclusions cannot be drawn from a case study, let alone resolution of the continuing controversy of whether EHS is a ‘real’ disease. What we provide here is a road map of a possible path forward, by identifying potentially crucial cellular features to investigate in the large cohorts of EHS patients that will be needed to achieve statistically meaningful conclusions.

Nonetheless, our hypothesized mechanism for EHS indeed plausibly fits with all of the known features of this syndrome. The broad and poorly defined range of symptoms, the varying backgrounds and unrelated case histories of these patients, and the lack of any standardized biochemical markers for EHS can all be explained by an immune response triggered by cellular byproducts of ROS and oxidative stress. Because EMFs directly induce cellular ROS, even trace increase such as caused by exposure to household telecommunications could be sufficient to trigger debilitating immune reactions, especially to exposed oxidized lipid or protein byproducts in the membranes of the vascular system. Such a mechanism would also imply a pronounced genetic contribution to EHS. For example, genetic conditions resulting in compromised, hyperactive immune systems; reduced physiological protective mechanisms for oxidative stress; or even in differential susceptibility of tissues and organs to oxidative damage and/or auto-antibodies could help explain why only a few persons in the population end up with EHS.

The good news is that this hypothesis is fully testable. Further research should include analysis of antioxidant mechanisms in cells and organs of EHS patients as compared to healthy controls; genome sequencing to detect anomalies in families with a history of EHS; rigorous characterization of immune response to byproducts of oxidative stress in EHS patients; and full characterization of ROS and redox-related modulation in different cell types and organs in response to EMF exposure in susceptible patients. All of these approaches are within technological reach and should resolve the pathophysiological basis of EHS.

Potential therapeutic implications of this study include the possibility of developing diagnostics to screen ESH susceptible individuals before full-blown symptoms can develop (prevention). Further potential avenues for EHS therapy would include the development of novel anti-oxidant pharmaceuticals, which may prevent the acute symptoms caused by transient spikes in oxidative stress induced by exposure electromagnetic fields. Alternatively, further research into noninvasive methods that down-regulate ROS (such as Photobiomodulation Therapy) may prove promising. Although the limited exposure to PBM did not provide a cure in this patient (Figure 4), it helped alleviate some of his milder symptoms, suggesting research into optimizing the dose parameters might prove effective. Finally, therapies directly targeting the immune response may be developed to improve tolerance against specific antigens induced by exposure to electromagnetic fields.

In sum, this study presents the case report of a patient with severe intolerance to EMF presenting with reduced levels of cellular anti-oxidants and significant increase in antibodies to cellular byproducts (LDLox) of oxidative stress. Since it has been demonstrated that man-made EMF sources (telecommunications, magnetic fields) induce increased levels of cellular oxidants, we hypothesize that EHS symptoms are consistent with a hypersensitive immune reaction to the byproducts of cellular oxidative stress. We hope in this way to inform research efforts toward unraveling the underlying basis and eventual treatment of this puzzling syndrome. We end with the recommendation that the subject of this case report, a young man of 25 years, may be an ideal subject both for studies of the root causes of EHS, for genetic studies of EHS lineages, and to participate in clinical trials of novel therapeutic strategies to help find a cure.

## Data Availability

All data in the manuscript are available from the corresponding author upon request.
